# Risk factors of frailty and functional disability in community-dwelling older adults: a cross-sectional analysis of the FREEDOM-LNA cohort study

**DOI:** 10.1186/s12877-022-03447-z

**Published:** 2022-09-19

**Authors:** Achille Tchalla, Cécile Laubarie-Mouret, Noëlle Cardinaud, Caroline Gayot, Marion Rebiere, Nathalie Dumoitier, Karen Rudelle, Michel Druet-Cabanac, Marie-Laure Laroche, Sophie Boyer

**Affiliations:** 1 Laboratoire VieSanté - UR 24134 (Vieillissement, Fragilité, Prévention, e-Santé), Institut OMEGA HEALTH, Université de Limoges, 87000 Limoges, France; 2 CHU de Limoges, Pôle HU Gérontologie Clinique, 2 Avenue Martin-Luther King, 87042 Limoges, France; 3 Unité de Recherche Clinique Et d’Innovation (URCI) en Gérontologie, CHU de Limoges, Pôle HU Gérontologie Clinique, Limoges, France; 4 Department of Clinical Geriatric, University Hospital Centre, 2 Avenue Martin Luther King, 87042 Limoges, France; 5 Département de Médecine Générale, Faculté de Médecine de Limoges, Limoges, France; 6 Centre de Pharmacovigilance Et de Pharmaco-Épidémiologie, CHU de Limoges, Limoges, France

**Keywords:** Aging, Dependence, Disability, Frailty, Physical functioning

## Abstract

**Background:**

Frailty is a geriatric syndrome associated with disability and negative health outcome. To determine the factors associated with frailty and functional disability in older participants living in community in France. We included 753 community-dwelling old participants with available frailty data at baseline.

**Results:**

Overall, 31.9% were frail, 58.3% were prefrail, and 9.8% were robust. The SMAF (French acronym for Functional Autonomy Measurement System) score was significantly lower (mean ± standard deviation: -25.8 ± 11.2) in frail participants compared to prefrail (-14.3 ± 9.7) or robust participants (-8.1 ± 7.0); 82% of frail older participants had limitation in at least one ADL and 97.5% in at least one IADL compared to 54.2 and 76.8%, respectively of pre-frail and 29.7 and 47.3% of robust participants. Age, depression, impaired cognition and diabetes were significantly associated with higher odds of frailty. These variables were also strongly associated with functional disability. Female gender, polypharmacy, and smoking were additional variables significantly associated with degraded SMAF and/or ADL/IADL.

**Conclusions:**

This study showed that functional disability increased proportionally to frailty, and depression, cognitive decline and diabetes are modifiable risk factors significantly associated with frailty and functional disability.

## Introduction

Life expectancy has increased worldwide and consequently there is a need for integrated care to maintain aging population in a good health, high mental and physical function, well-being, and social engagement and productivity for longer time [1]. Aging is a heterogeneous process with high variability in health status and disability between individuals. Older individuals may be roughly classified in 3 categories i.e. peoples in good health with stable functional status, frail participants with loss in their ability to withstand disease without loss of function, and dependent individuals with functional physical or mental decline [2, 3].

Dependence occurred when functional capacities to perform basic day-to-day activity to take care of oneself such as dressing, washing, eating, moving, or using the washroom are lost [4]. By definition, loss of independence (i.e. disability) is a multidimensional process that results from the interaction between health conditions and other personal characteristics (age, sex, educative level, etc.…) and social and environmental factors. Disability is commonly measured by self-reported difficulties and/or inability to develop activities of daily living (ADL) and instrumental activities of daily living (IADL). In France, the Functional Autonomy Measurement System (SMAF) is also widely used to assess functional autonomy of older adults [5, 6]. The SMAF combines measurements in ADL and IADL limitation, but also in mobility, communication (seeing, hearing, speaking), and mental function limitation and thus is more complete instrument to predict disability and loss of independence. Its validity, reliability and sensitivity to change show very good ratings compared to other instruments used in older populations [7].

Frailty is described as an intermediate, reversible status between healthy aging and dependence [2]. It is considered as a major risk for adverse outcome in older subjects and frailty prevention is believed to be a crucial indicator of successful aging [8]. Frailty has been defined as a clinical syndrome in which a decreased reserve and resistance to internal or external stressors, resulting from cumulative decline across multiple physiological systems, increases vulnerability to adverse outcomes (e.g. confusion, depression, falls, malnutrition) [9]. As a consequence, frail older people are at increased risk of incident disability and dependence [10]. Frailty can be simply diagnosed by assessing the limitation in three or more of five conditions (Fried’s criteria) including slowness, weakness, exhaustion, low activity, and weight loss [9]. In addition to these physical signs and symptoms, there are other potentially important components of the frailty syndrome to be considered in older subjects such as cognition, mood, sensory impairments, social and economic factors [2, 11]. Overall, there is a considerable overlap between comorbidity, frailty and disability in community-dwelling older subjects [12, 13]. It is thus of importance to determine the factors associated with frailty and disability in this population. This should help to implement preventive measures to avoid decline in functional capacity and dependence. Geriatric units specialized in evaluation, management and prevention of disability in frail population are helpful to promote the quality of life of older people and increase life expectancy without disability [14]. In our institution, we followed a regional longitudinal cohort of community-dwelling older subjects (FREEDOM-LNA). This cohort was composed of participants s relatively aged (mean 84 yrs, 68% of women), of whom more than 30% were frail and more than 50% presented signs of dependence [15]. In the present cross-sectional study, we analysed the association between frailty and disability and determined factors associated with frailty and disability among participants of the FREEDOM-LNA cohort composed of community-dwelling older adults who were interested to receive a comprehensive geriatric assessment at home.

## Materials and methods

### Study design

This cross-sectional study was carried out using data from the FREEDOM-LNA Cohort. Briefly, the FREEDOM-LNA longitudinal study was an observational study conducted by the UPSAV at the University Hospital of Limoges, France. The UPSAV is a preventive health service to help robust or frail people with the aim for maintenance at home. Overall 1085 community-dwelling subjects over 75 years, or over 65 years with at least two comorbidities were included. Detailed characteristics of the FREEDOM cohort have been reported previously [15].

The study protocol was reviewed and approved by the local Institutional Review Board (CEREES, Limoges; Approval number: TPS 429,669) and by the French Data Protection Authority (CNIL) insuring protection of individualized data according to the French law. Informed consent for data processing was obtained from all subjects (or legal representatives). All procedures were carried out in accordance with the Helsinki Declaration and its later amendments.

### Measurement of frailty

Frailty was assessed using the Fried criteria [9] including weakness as assessed by grip strength of the dominant hand < 20%, slowness (walking speed < 20% of normal), low level of physical activity (< 20% of energy expenditure), low energy or self-reported exhaustion, and unintentional weight loss (4 to 5 kg since the previous year). Participants were considered as frail when at least 3 criteria were present, pre-frail when there was one or two criteria and robust were there was no criterion.

### Measurement of functional disability

This study included two widely used questionnaires of self-reported measures of self-care tasks administered by the study health professional (a geriatric physician or trained nurse). The ADL disability measure focused on the ability to perform six essential self-care tasks: bathing, dressing, eating, showering, toileting, and getting out of bed to chair [16]. The IADL focused on the ability to perform seven household tasks: using phone, grocery shopping, preparation of meals, housekeeping, doing laundry, taking care of medication, and managing finances [17].

Disability was also assessed using the SMAF (French acronym for Functional Autonomy Measurement System) questionnaire [5]. The SMAF is a validated 29-item (87 questions) standard questionnaire based on the World Health Organization (WHO) classification of disablement (International Classification of Impairments, Disabilities and Handicaps).

### Cognitive capacity and depression

The cognitive capacity was measured using the Mini Mental State Examination (MMSE) screening test [18]. Subjects were considered to have a cognitive deficit if MMSE score was < 24 adjusting for education (≤ 20 individuals with low education, ≤ 23 in subjects with medium education and ≤ 26 in individuals with a high education). Depression over the past week was monitored using the Geriatric Depression Scale (GDS) as described previously [19]. GDS scores ranging from 0 to 5 were indicative of normal mood; scores between 5 and 9 of a risk of depressive symptoms, and scores > 9 of severe depressive symptoms.

### Covariates

Covariates that were deemed to influence the frailty and functioning capacity were measured. This included sociodemographic variables (age, sex, and education), cardiovascular risk factors (hypertension, dyslipidaemia, diabetes, obesity) polymedication (defined as at least five medications per day), lifestyle (smoking, alcohol consumption, and living arrangement), body mass index in three classes (< 18; 18–21; ≥ 21 kg/m^2^), cognitive impairment (MMSE < 24 adjusting for education), and depressive symptoms (GDS > 9).

### Statistical analysis

All variables were described using mean and standard deviation for continuous variables and percentages for categorical variables. No imputation was carried out for missing values.

Univariate analyses were performed using linear regression models to determine the association of each covariate with the SMAF score (as a quantitative variable) and using logistic regression models to determine the association of each covariate with the presence of at least one ADL, with at least one IADL, or with frailty (frail versus non-frail). Multivariate regression models were implemented to determine independent covariates. All factors significantly associated with impaired functional disability or with frailty at the 20% level were included in the final model. We applied a backward stepwise selection controlled for all factors with a *p*-value < 0.20 in univariate models to select only the significant factors (at the 5% level) and kept the confusion factors. The final model was adjusted on socio-demographic and health-related covariates. Linear regression coefficients and odds ratio (OR) were estimated with their 95% confidence interval (95%CI).

The association between functional disability and frailty was described in contingency tables and tested using a Chi-squared test or an exact Fisher test. The ADL, IADL, and SMAF were described in each group of frailty and compared between groups using a Kruskal–Wallis test (SMAF) or chi-squared test (ADL and IADL). All tests were bilateral and considered as significant at the alpha level of 0.05 (*p* < 0.05).

## Results

Analyses were performed in 753 participants with available frailty and functional capacity data. Main baseline characteristics in these patients are described in Table 1. Overall, 240 (31.9%) participants were frail, 439 (58.3%) were pre-frail and 74 (7.8%) were robust. The frequent frailty criteria in this cohort were low grip strength (80.1% of participants), low physical activity (55.5%), and low walking speed (31.9%). Functional limitation in at least one ADL was present in 456 (60.6%) subjects and limitation in at least one IADL in 605 (80.5%) participants.

**Table 1:** Main characteristics of the study population (*N* = 753)

**Characteristics**		**N (%)**
Age	Mean ± SD	83.1 ± 5.8
Sex	Male Female	244 (32.4) 509 (67.6)
Education^a^	Low Medium High	451 (60.0) 127 (16.9) 174 (23.1)
Living arrangement	Living alone	405 (53.9)
Cardiovascular morbidities	Hypertension Dyslipidaemia Obesity Diabetes Smoking Alcohol	548/735 (74.6) 363/733 (49.5) 197/729 (27.0) 151/732 (20.6) 100/731 (13.7) 28/732 (3.8)
Polymedication	≥ 5 medications / day	599/738 (81.2)
Nutritional status (MNA)	Mean ± SD	24.2 ± 3.7
Functional status	ADL IADL	5.3 ± 0.9 5.7 ± 2.0
	SMAF	-17.3 ± 11.6
Depressive symptoms	GDS > 9	302/634 (47.6)
Cognitive deficit	MMSE < 24	245/712 (34.4)
FRIED criteria	Weight loss Low energy/exhaustion Low grip strength Low walking speed Low physical activity	82 (10.9) 142 (18.9) 603 (80.1) 240 (31.9) 418 (55.5)
Frailty	Frail Pre-frail Robust	240 (31.9) 439 (58.3) 74 (9.8)

^a ^Low: primary certificate level; Medium: Middle school, High: Secondary or high school

**Fig. 1:**
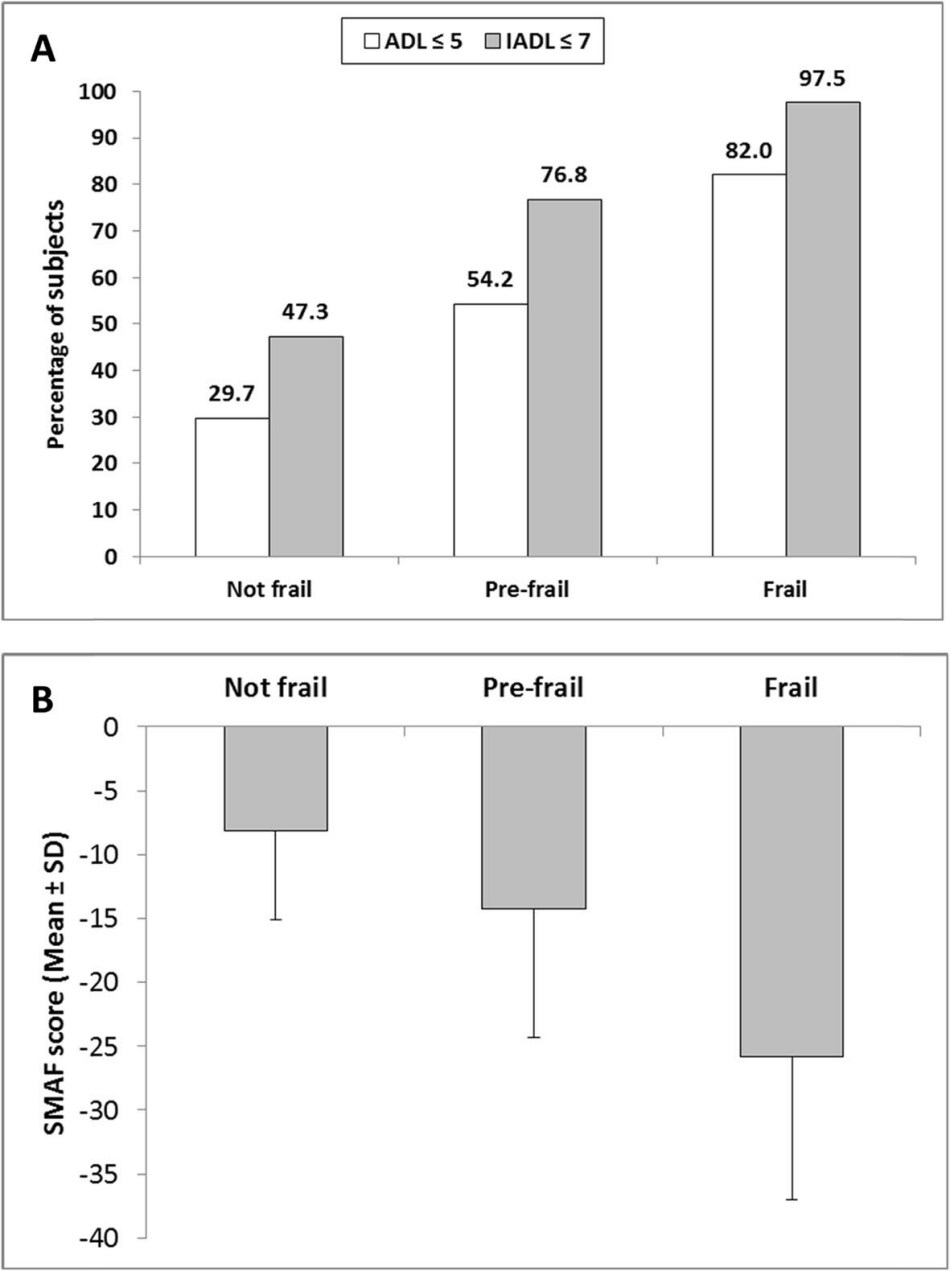
Relationship between frailty and loss of autonomy (ADL, IADL, and SMAF). **A** Loss of independence in functional activities in ADL and IADL according to frailty (*N* = 752); (**B**) SMAF score according to frailty (*N* = 747); a SMAF score between 0 and -7 indicates complete autonomy, between -8 and -14 average autonomy, and a SMAF score ≥ -15 a moderate to severe loss of autonomy

As shown in Fig. 1A, limitation in at least one ADL was present in 29.7, 54.2 and 82.0% of the robust, pre-frail and frail participants, respectively (*P* < 0.0001). Limitation in at least one IADL was present in 47.3, 76.8 and 97.5% of the non-frail, pre-frail and frail participants, respectively (*P* < 0.0001). Consistently, the mean SMAF score was significantly lower (*p* < 0.0001) in frail participants (-25.8 ± 11.2) compared to pre-frail participants (-14.3 ± 9.7) or non-frail participants (-8.1 ± 7.0) (Fig. 1B).

### Factors associated with frailty

In univariate logistic regression, the covariates significantly associated with frailty (frail vs. non-frail) were age (*P* < 0.0001), polymedication (*P* = 0.007), diabetes (*P* = 0.013), a GDS score < 9 (*P* < 0.0001) and a cognitive deficit MMSE (< 0.0001). In multivariate analysis, factors significantly associated with higher odds of frailty were age (OR = 1.08), GDS > 9 (OR = 4.20), a cognitive deficit MMSE (OR = 1.94) and diabetes (OR = 1.81), while living alone was inversely associated with frailty (OR = 0.63, *P* = 0.0214) (Table 2).

**Table 2:** Factors associated with frailty in multivariate logistic regression analysis

Frailty (*N* = 582) *	Adjusted OR [95% CI]	*P* value
Age (continuous variable)	1.08 [1.04; 1.12]	< 0.001
Depressive symptoms, GDS > 9 (Yes vs. No)	4.20 [2.81; 6.29]	< 0.001
Cognitive deficit, MMSE < 24 (Yes vs. No)	1.94 [1.30; 2.91]	0.001
Diabetes (Yes vs. No)	1.81 [1.13; 2.89]	0.013
Living alone (Yes vs. No)	0.63 [0.42; 0.93]	0.021

*N* = 582 (171 subjects had at least one covariate missing); OR: odd ratio (frail versus non-frail)

### Factors associated with loss of autonomy

In univariate logistic regression, the covariates significantly associated with the SMAF as a continuous variable were age (*P* < 0.0001), educational level (*P* = 0.0003), living alone (*P* < 0.0001), smoking (*P* = 0.0401), body mass index in 3 classes (< 18, 28–21, ≥ 21 kg/m^2^) (*P* = 0.0022), polypharmacy (*P* < 0.0001), diabetes (*P* = 0.0007), dyslipidaemia (*P* = 0.0407), a GDS > 9 (*P* < 0.001), and a pathologic MMSE (*P* < 0.0001). Other variables with a *P* value < 0.20 were sex (*P* = 0.1181), hypertension (*P* = 0.0641), and alcohol consumption (*P* = 0.1554).

As shown in Table 3, independent factors significantly associated with the SMAF score using the multivariate linear regression model were age (-0.49), a pathologic MMSE (-10.42), a GDS score > 9 (-4.93), diabetes (-3.58), polymedication (-3.50), smoking (-3.23), and education (-3.22). Living alone showed an inverse association with the risk of SMAF (2.30).

**Table 3:** Factors associated with SMAF score

SMAF score (*N* = 707) *	Linear regression coefficient (SD)	*P*-value
Age (continuous variable)	-0.49 (0.06)	< 0.001
Cognitive deficit, MMSE (Yes vs. No)	-10.42 (0.78)	< 0.001
Depressive symptoms, GDS > 9 (Yes vs. No)	-4.93 (0.73)	< 0.001
Diabetes (Yes vs. No)	-3.58 (0.91)	< 0.001
Polymedication ≥ 5 (Yes vs. No)	-3.50 (0.99)	< 0.001
Smoking (Yes vs. No)	-3.23 (1.06)	0.002
Education level (medium vs. high)	-3.22 (0.89)	< 0.001
Living alone (Yes vs. No)	2.30 (0.74)	0.002

^*^ 46 subjects had at least one missing covariate

Factors significantly associated with limitation in ADL or IADL in multivariate regression models are described in Table 4. Factors significantly associated with higher odds in limitation of at least one ADL were age (OR = 1.06), polymedication (OR = 1.87), a pathologic MMSE (OR = 1.57), and a GDS > 9 (OR = 1.54), while male gender was inversely associated with limitation in ADL (OR = 0.47).

**Table 4:** Factors associated with ADL and IADL in multivariate logistic regression analysis

	**Adjusted OR [95% CI]**	***P* value**
Limitation in ADL (ADL ≤ 5) *		
Age (continuous variable)	1.06 [1.03; 1.09]	< 0.001
Polymedication ≥ 5 (Yes vs. No)	1.87 [1.23; 2.85]	0.004
Cognitive deficit, MMSE < 24 (Yes vs. No)	1.57 [1.10; 2.22]	0.012
Depressive symptoms, GDS > 9 (Yes vs. No)	1.54 [1.11; 2.12]	0.009
Sex (Males vs. females)	0.47 [0.34; 0.66]	< 0.001
Limitation in IADL (IADL ≤ 7) *		
Age (continuous variable)	1.12 [1.08; 1.17]	< 0.001
Cognitive deficit, MMSE < 24 (Yes vs. No)	7.79 [4.12; 14.75]	< 0.001
Polymedication ≥ 5 (Yes vs. No)	3.53 [2.06; 6.02]	< 0.001
Diabetes (Yes vs. No)	2.87 [1.57; 5.26]	0.007
Educational level (low vs. high)	2.22 [1.34; 3.68]	0.002
Depressive symptoms GDS > 9 (Yes vs. No)	1.82 [1.17; 2.83]	0.008
Dyslipidaemia (Yes vs. No)	0.50 [0.32; 0.80]	0.003

^*^ ADL: *N* = 712 (41 participants had at least one covariate missing); IADL: *N* = 707 (46 participants had at least one covariate missing)

Factors significantly associated with higher odds in limitation of at least one IADL were age (OR = 1.12), a cognitive deficit MMSE (OR = 7.79), polypharmacy (OR = 3.52), diabetes (OR = 2.87), poor education (OR = 2.22), a GDS > 9 (OR = 1.82), while dyslipidaemia was inversely associated with limitation in IADL (OR = 0.50).

## Discussion

### Main results and study reporting similar results

In this study, we analysed various covariates including demographic variables, comorbidities, cognitive and emotional variables which could predict the risk of frailty or loss in functional capacities in older adults of the FREEDOM-LNA cohort. This cohort was composed of participants > 75-year old or between 65 and 75 yrs with at least one morbidity. Consequently, the prevalence of frailty using Fried’s criteria was quite higher (32%) than the prevalence reported in other cross-sectional studies (around 10%) in community dwelling old adults [9, 20–22]. Nevertheless, high frailty rates between 20 and 30% have also been reported in other studies in France [23] or Spain [24].

In our multivariate regression analysis, age was an independent predictor of frailty, consistent with higher physical frailty in the oldest old [25]. Other factors positively associated with higher odds of frailty in this cohort were cognitive impairment, depressive symptoms, and diabetes. Contrary to other studies, sex and educational level were not significant factors associated with frailty when adjusted for other covariates [20, 24, 26]. In addition, we found that living alone was inversely associated with frailty, which was also reported in another study [27].

The results of the present study are similar to those of others showing an association between frailty and depression and/or cognitive impairment [27–29]. Depressive symptoms as assessed by a GDS score > 9 was the variable with the strongest association with frailty, with and odd ratio of 4.2. This result is consistent with other cross-sectional studies showing that old participants with depression were at approximately fourfold increased odds of having frailty [30]. As reviewed by Kok et al. [31], depression in geriatric participants is frequent but it is difficult to know whether frailty is a comorbidity, cause or consequence of depression. There are various factors associated with depression in the older population including intrinsic factors (personality traits, functional impairment) and/or extrinsic factors (social isolation, stressful life events). Nevertheless, common pathophysiological alterations have been proposed including hormonal changes and low-grade inflammation as reviewed by Buigues et al. [32].

In our cohort, frailty was also positively associated with decline in cognition as assessed using the MMSE score, consistently with other cross-sectional studies [28, 33]. Some interrelations probably exist between frailty and cognition in older participants including decrease in food intake, weight loss and sarcopenia. In another study, this association was shown to be independent of confounding factors such as age, gender, educational level, medical history of hypertension, diabetes, stroke and metabolic syndrome or nutritional status [33].

In our cohort, diabetes was present in 21% of participants and the odd of frailty was increased in diabetic patients, independently of age, depression or cognitive decline. Diabetes has been previously found to be associated with increased frailty in older people [34], and faster increasing frailty trajectory compared to older adults without diabetes [35]. It appears that diabetes and frailty share some pathophysiological mechanisms such as low-grade inflammation, insulin resistance and sarcopenia [36, 37]. Diabetes is also a major risk factor of cardiovascular diseases and has been associated with depression [38].

Frailty is the main risk factor for functional disability in 60 + old adults [39]. Our study showed that functional disability was proportionally higher in frail and prefrail participants compared to robust participants. Overall, regardless of frailty, the proportions of participants with limitations in at least one ADL or IADL was much higher compared to other studies in community-dwelling older adults which may be explained by sociodemographic and health characteristics across the different studies [25, 40]. We identified seven independent factors significantly associated with functional disability as assessed using the SMAF score i.e. age, impaired cognition, depression, diabetes, polymedication, smoking and education. Age, depression, cognitive impairment and diabetes were also independent predictors of frailty, as discussed above, which suggests considerable interrelation between frailty and functional disability. Age, polypharmacy, cognitive impairment and depressive symptoms were common risk factors for IADL and ADL, while diabetes and education were only significant risk factors for IADL and female gender only for ADL. Subjects in this cohort were proportionally more affected in IADL than in ADL activities, and odds ratios were stronger for IADL than for ADL.

### Strengths of the study

The strongest predictors of functional disability as assessed by SMAF or IADL were age, cognitive impairment, depressive symptoms, polypharmacy and diabetes. This result is in lines with previous studies suggesting that decline in cognitive and emotional capacities are important factors to explain loss of autonomy in community-dwelling older subjects [28]. A meta-analysis of studies confirmed that diabetes increased the risk of physical disability [41]. Polypharmacy in older people was also previously reported as an independent factor associated with impaired functional ability and cognitive function [42].

Taken together, our study suggests interventional measures to reduce frailty, loss of independence, and disability in older adults. Screening of frailty and management should be a clinical priority especially in old diabetic patients as well as in patients with depression and cognitive dysfunction [37]. Physical activity, depressive symptoms, and cognitive impairment have been suggested as potentially modifiable mediators [43]. This could be successfully addressed using appropriate non-pharmacological measures, including regular physical activity [31]. Exercise-based interventions in older adults may be beneficial by increasing confidence, self-esteem, positive behaviour, and social relationship [44]. Although early identification and intervention are recommended, some exercise intervention programs may be useful since they have been shown to reverse frailty and improve cognition emotion and social networking in community-dwelling frail older adults [45]. For diabetic patients, interventions to prevent frailty using nutrition and exercise training are required [34, 37]. Sarcopenia has been considered as the most important target for the management of frailty in diabetic patients [36]. The risk–benefit of pharmacological interventions and targets for glucose control should be discussed. As reported previously, our results confirmed that polymedication is an independent risk factor for frailty and disability, and a recent interventional study showed that reducing polypharmacy in frail older subjects improved depression, mental health status, function and frailty [46].

### Limitations of the study

This study has some limitations to be mentioned. This study enrolled relatively old participants (> 80 yrs on average) with high prevalence of frailty and functional disability, and thus conclusions may be inappropriate to young older subjects 65–75 yrs. However, a strength of this study is the relatively high number of participants included with various frailty criteria and functional disability to explore predictive factors with sufficient statistical power. As a cross-sectional study, it is not possible to determine if the associated factors are the cause or consequence of frailty or disability. Depression and cognitive impairment may have reciprocal relationship with frailty or functional disability [30]. We did not investigate other factors which may be associated with frailty such domestic environment [24], socio-economic status (income) [47] or previous adverse outcomes such as falls and hospitalisations [26].

### Advantages of this study

This study showed that functional disability increased proportionally to frailty, and depression, cognitive decline and diabetes are modifiable risk factors significantly associated with frailty and functional disability in older population. This cohort was composed of community-dwelling older adults who were interested to receive a comprehensive geriatric assessment at home. Thus, such assessment may be less considered in apparently healthy older people. Further clinical interventional studies are needed to identify medical and behavioural interventions for frailty, depression, and cognitive impairment that could prevent or limit functional disability. As the causal relationship is not established, longitudinal data analysis will be explored in upcoming reports to determine the trajectories of frailty and functional disability and the prognostic association with clinical outcome including falls, depression, hospitalisation, comorbidity, and mortality.

## Data Availability

“Doctor Sophie Boyer, PhD (sophie.boyer@chu-limoges.fr) who should be contacted if someone wants to request the data.”

## Abbreviations

ADL: Activity of Daily Living
AGGIR: Instrument for evaluating dependency in elderly in France
CGA: Comprehensive Geriatric Assessment
EQ-5D: EuroQol-5 Dimension
FRIED’S CRITERIA: Physical frailty
FREEDOM-LNA cohort: French acronym for Frailty, Clinical Research and Evaluation at Home in Limousin – Nouvelle Aquitaine
GDS: Geriatric Depression Scale
IADL: Instrumental Activities for Daily Living
MMSE: Mini Mental State Examination
MNA: Mini Nutritional Assessment
SMAF: Functional Autonomy Measurement system
SPPB: Short Physical Performance Battery
UPSAV: Geriatric mobile team (Unité de Prévention de Suivi et d'Analyse du Vieillissement)
